# Catalytic and biocatalytic degradation of microplastics

**DOI:** 10.1002/EXP.20230018

**Published:** 2023-12-19

**Authors:** Mohamad Zandieh, Erin Griffiths, Alexander Waldie, Shuhuan Li, John Honek, Fereidoun Rezanezhad, Philippe Van Cappellen, Juewen Liu

**Affiliations:** ^1^ Department of Chemistry University of Waterloo Waterloo Ontario Canada; ^2^ Waterloo Institute for Nanotechnology University of Waterloo Waterloo Ontario Canada; ^3^ Water Institute University of Waterloo Waterloo Ontario Canada; ^4^ Ecohydrology Research Group Department of Earth and Environmental Sciences University of Waterloo Waterloo Ontario Canada

**Keywords:** catalysis, microplastics, nanozymes

## Abstract

In recent years, there has been a surge in annual plastic production, which has contributed to growing environmental challenges, particularly in the form of microplastics. Effective management of plastic and microplastic waste has become a critical concern, necessitating innovative strategies to address its impact on ecosystems and human health. In this context, catalytic degradation of microplastics emerges as a pivotal approach that holds significant promise for mitigating the persistent effects of plastic pollution. In this article, we critically explored the current state of catalytic degradation of microplastics and discussed the definition of degradation, characterization methods for degradation products, and the criteria for standard sample preparation. Moreover, the significance and effectiveness of various catalytic entities, including enzymes, transition metal ions (for the Fenton reaction), nanozymes, and microorganisms are summarized. Finally, a few key issues and future perspectives regarding the catalytic degradation of microplastics are proposed.

## Graphical abstract

In this article, the catalytic degradation of microplastics using various catalysts including enzymes, small molecule catalysts, microbes, and nanozymes is reviewed. Practical environmental considerations, definition of degradation, sample preparation, and product characterization are also discussed.

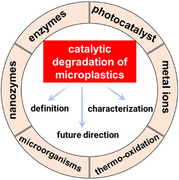

## 1 INTRODUCTION

Low price, light weight, versatile manufacturing, and chemical stability have made plastic materials desirable for a wide variety of applications, including food conservation,^[^ ^1^ ^]^ textiles,^[^ ^2^ ^]^ building construction,^[^ ^3^ ^]^ and the medical industry.^[^ ^4^ ^]^ As a result, annual production of plastic materials has surpassed 300 million metric tons in the last couple of years.^[^ ^5^ ^]^ Plastic materials are extremely resistant to biodegradation, and the natural degradation of plastics can take up to several hundreds of years.^[^ ^6^ ^]^ The most conventional methods to handle plastic waste are landfilling and incineration, which are major sources of environmental pollution.^[^ ^7^, ^8^ ^]^

Microplastics are submillimeter plastic particles that are either intentionally manufactured to be used in cleaning and cosmetic products (primary microplastics) or generated in the environment when bigger plastic wastes are fragmented via photochemical, chemical, mechanical, and/or biological transformations (secondary microplastics).^[^ ^9^, ^10^ ^]^ Owing to their large surface‐to‐volume ratio, microplastics in the ecosystem sorb and transport a variety of hazardous contaminants, including heavy metals,^[^ ^11^ ^]^ persistent organic pollutants,^[^ ^12^ ^]^ and pathogenic species.^[^ ^13^, ^14^ ^]^ Therefore, microplastics pollution of the food chain poses a serious threat to human health potentially stimulating a variety of diseases from disruption of the immune system to cancer.^[^ ^15^, ^16^ ^]^

The most commonly detected plastic particles in environmental waters are, respectively, polyethylene (PE), polypropylene (PP), polystyrene (PS), polyvinyl chloride (PVC), and polyethylene terephthalate (PET). The order of abundance is due to both the production demand of the polymers as well as their densities.^[^ ^17^ ^]^ Microplastics not only are considered a contamination source of water and sediments, but their interaction with other pollutants also interferes with and complicates common water and wastewater treatment processes such as coagulation, air flotation, and membrane filtration.^[^ ^18^ ^]^

Different techniques for microplastics separation from water and sediment have been reported such as sorption on microalgae,^[^ ^19^, ^20^ ^]^ membrane filtration,^[^ ^21^ ^]^ magnetic separation,^[^ ^22^, ^23^ ^]^ and density separation.^[^ ^24^ ^]^ Methods for recycling or degrading microplastics are desirable. In particular, the catalytic degradation of microplastics is an area of active research that explores various catalysts and reaction conditions to develop effective and sustainable methods for breaking down plastic particles into smaller entities that are less persistent and harmful.

Catalytic degradation of bulk plastic wastes has been elaborated in a few review articles.^[^ ^25^, ^26^, ^27^ ^]^ For example, in a recent paper, Chen et al. discussed the application of catalytic technologies, including thermo‐, electro‐, bio‐, and photocatalysis for the recycling of waste plastic resources.^[^ ^27^ ^]^ However, micro/nanoplastics need to be viewed independently of bulk plastics. On one hand, owing to the high surface‐to‐volume ratio of microplastics, they are more susceptible to chemical and biological degradation than bulk plastics. On the other hand, the detection and collection of microplastic waste is far more challenging than that of bulk plastics in the environment. Therefore, it is vital to dedicate attention to the research of catalytic degradation of microplastics as well. Efremenko et al. recently reviewed the catalytic degradation of microplastics, with a focus on the chemical aspect of catalytic processes.^[^ ^28^ ^]^

In the present study, we review the catalytic and biocatalytic degradation of microplastics from a novel viewpoint. First, we critically discuss the definition of microplastic degradation and methods for characterizing degradation. Then, the criteria for sample preparation and the reaction conditions for an efficient degradation experiment are reviewed. Next, the catalytic degradation of microplastics using various catalytic entities, including natural enzymes, transition metal ions (for Fenton chemistry), nanozymes, and microorganisms, is discussed. Finally, a few future research directions are proposed.

## 2 DEFINITION AND CHARACTERIZATION OF DEGRADATION

In the literature on microplastics, degradation appears to be a broadly used term. From an environmental standpoint, it is desirable that plastic polymers ultimately transform into high‐value products, such as methane, formate, C_2_ fuels, and acetic acid, or benign by‐products, such as water and carbon dioxide.^[^ ^29^ ^]^ However, not all the studies on the “degradation of microplastics” in the literature achieved this goal.

**FIGURE 1:**
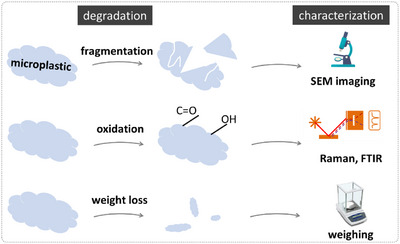
Schemes depicting various definitions of microplastics degradation in the literature. The characterization methods for each definition include fragmentation (SEM imaging), oxidation (Raman, FTIR), and weight loss (weight loss measurement).

Depending on the definition of degradation employed, various characterization techniques can be used (Figure 1). The most common techniques reported in the literature include physical analyses (such as scanning electron microscopy [SEM] and weight loss) and chemical analyses (such as Fourier‐transform infrared spectroscopy [FTIR], Raman spectroscopy, and mass spectrometry).^[^ ^30^ ^]^ In the literature review of microplastics, degradation is referred to in different categories as follows:

### 2.1 Fragmentation

The initial stages of the degradation processes can result in the breakdown of chemical bonds within the plastic polymer chains, which can primarily appear in the form of cracks and cavities in microplastic materials. Upon further generation of cracks and weakening of the polymer chains, the plastic material becomes more susceptible to mechanical stress, which can ultimately lead to the fragmentation of microplastic particles into smaller nano‐ and microplastics. Many studies referred to the fragmentation of microplastics as degradation.^[^ ^31^, ^32^, ^33^, ^34^ ^]^ Moreover, particle size distribution analysis can provide information on the range of fragment sizes. Microscopic analyses are most effective with previous information on the samples, such as conducting these techniques on microplastics before degradation experiments to compare the results with those of degraded plastic samples.^[^ ^35^ ^]^

### 2.2 Oxidation

Oxidation reactions are essential for the degradation of microplastics. Enzymes can facilitate the breakdown of microplastic polymers by introducing oxygen‐containing functional groups (e.g., hydroxyl and carbonyl groups) into polymer chains, and oxidative cleavage of polymer chains can result in the degradation of microplastics. Similarly, photocatalysts generate reactive oxygen species^[^ ^36^, ^37^ ^]^ that attack the polymer chains of microplastics, initiating oxidation reactions that break down the plastic structure. Therefore, many studies examine the oxidation of microplastics to account for degradation.^[^ ^38^, ^39^, ^40^ ^]^ Spectroscopic methods, such as FTIR and Raman spectroscopy, can provide valuable information regarding the oxygen‐containing functional groups caused by mechanisms of degradation.^[^ ^41^ ^]^ These methods are sensitive to changes on the polymer surface and polymer additives, making them ideal for polymer composition determination but have limited use for colored plastics and environmental plastics that are not cleaned.^[^ ^34^ ^]^ X‐ray photoelectron spectroscopy (XPS) can also be used to analyze the content of oxygen and carbon atoms to quantify the extent of oxidation. Moreover, because oxidation can deteriorate the thermal stability of microplastics,^[^ ^42^ ^]^ methods such as thermogravimetric analysis (TGA) can be used to provide insights into the degree of oxidation of microplastics.

### 2.3 Weight loss

Ultimately, complete degradation of microplastics, which is more favorable, is achieved when microplastics are degraded to the point where their polymer chains are fully broken down. The resulting fragments or monomers are further transformed into simple, naturally occurring, carbon‐containing compounds through biological or chemical reactions. Such degradation can be measured by simply assessing the solid weight loss during degradation. At the same time, chromatographic‐based methods such as liquid chromatography‐mass spectrometry and high‐performance liquid chromatography (HPLC) can be used to separate and identify organic degradation products. Biodegradation particularly helps with the upcycling of microplastics, where organic compounds derived from microplastic degradation can be further metabolized into high‐value products by microorganisms through various biochemical pathways.

## 3 PREPARATION FOR SAMPLE ANALYSIS

Before analysis of macro‐ and microplastics, such as degradation analysis, samples must be cleaned to avoid inconsistent results caused by contaminants on the plastic surface.^[^ ^43^ ^]^ The approaches and techniques used to clean samples are imperative to compare results reported in the literature, although plastic cleaning remains a somewhat enigmatic aspect of plastics research.^[^ ^44^ ^]^ Cleaning of samples depends on several factors including storage conditions of plastic samples, origin of the sample (field or lab sample),^[^ ^45^ ^]^ amount of debris on the sample, and durability and makeup of the polymer.^[^ ^46^, ^47^ ^]^

### 3.1 Origin of sample and polymer type

The origin of the sample provides insights into the level of physical breakdown of the polymer under environmental conditions,^[^ ^43^ ^]^ along with potential contaminants encountered.^[^ ^48^ ^]^ Environmental samples are more likely to undergo more mechanical breakdown and weathering than lab‐derived samples^[^ ^49^ ^]^ and encounter many substances that can leach into the polymer,^[^ ^48^ ^]^ leading to reduced purity levels and thus lower library matching rates with spectrometry analysis.^[^ ^50^ ^]^ The polymer type is typically known when samples originate from a laboratory environment, whereas field samples are commonly unknown, making them more difficult to clean effectively,^[^ ^43^ ^]^ because different polymer types have distinct cleaning methods. It is important to choose cleaning agents and protocols that have been well studied and are compatible with the plastic type, as many surfactants during the cleaning process cause changes in surface chemistry.^[^ ^51^ ^]^

### 3.2 Debris contamination

Debris contamination is a major issue when cleaning samples, as removal of debris may be difficult and require solutions that may alter or damage microplastics.^[^ ^46^ ^]^ The durability and properties of the polymer play a role in the solutions that can be used to remove debris, as mechanical methods for removing debris, such as microfiber cloth, leave micro‐scratches seen with SEM imaging.^[^ ^51^ ^]^

### 3.3 Cleaning chemicals

A popular polysorbate nonionic surfactant, Tween, which decreases hydrophobic interactions between polymer surfaces and contaminants, is often used to remove chemical coatings.^[^ ^52^ ^]^ Ethanol is another commonly used solution owing to its disinfectant properties and rapid evaporation.^[^ ^53^ ^]^ Popular methods to remove organic wastes attached to samples involve oxidative digestion by hydrogen peroxide paired with iron catalyst or alkaline digestion by potassium hydroxide.^[^ ^54^ ^]^

## 4 CATALYTIC DEGRADATION

Catalytic degradation of plastics was first reported 50 years ago, when Fields et al. utilized *Pullularia pullulans* microorganisms to secrete enzymes for the degradation of polycaprolactone.^[^ ^55^ ^]^ The research was then limited until the 2000s when plastic pollution was raised as a crisis.^[^ ^56^ ^]^ Since then, more than 500 publications have investigated particularly the catalytic degradation of “microplastics” by natural enzymes, transition metals, nanomaterials, and microorganisms (*Source*: www.scopus.com).

### 4.1 Degradation by natural enzymes

Enzymes are protein‐based catalysts that can accelerate reactions in biological systems. Unlike most conventional chemical catalysts, they act under mild aqueous conditions reducing the need for potentially toxic solvents and energy‐intensive heating. However, the labile nature of enzymes and their tendency to denature necessitate careful control of their conditions, preferably emulating their natural environment. Similarly, a substrate introduced into an enzyme should mirror its natural substrate in some regard. This is perhaps the greatest challenge in the field of enzymatic plastic degradation, as plastics have few if any natural analogs. Nevertheless, there have been successes, perhaps the greatest being the use of cutinase to depolymerize PET, a reaction that is beginning to be commercialized by biotech companies such as Carbios.^[^ ^56^ ^]^ Along with the depolymerization of PET, this section will focus on the enzymatic degradation of PE and PS, two carbon backbone plastics that still present a significant challenge. Together these three plastics compose more than half of annually discarded plastics and cover a variety of plastic types including hydrolyzable and non‐hydrolyzable plastics.^[^ ^57^ ^]^

#### 4.1.1 Enzymatic degradation of PET

The current body of literature on the enzymatic degradation of plastics focuses primarily on PET, a semi‐crystalline hydrolyzable plastic produced at a rate of ∼65.4 million metric tons per year (∼17% of all plastic).^[^ ^57^ ^]^ Originally considered a nondegradable polymer, PET biodegradation got its breakthrough moment when Müller et al. published that a cutinase from *Thermobifida fusca* caused the depolymerization and 50% weight loss of a commercial PET film.^[^ ^58^ ^]^ The chemical similarities between a plant's protective cutin and PET further stimulated the search for enzymes whose natural substrates mirror synthetic polymers. Since the discovery of cutinase for PET degradation, additional enzyme breakthroughs have come in the form of increased thermal stability above the *T*_g_ of PET (∼70–80°C). A more open active site enables the more efficient binding of PET and a greater acid tolerance since degradation products such as terephthalic acid acidify the solution.^[^ ^56^, ^59^ ^]^ These qualities lend themselves to industrial processes and allow for reduced substrate processing and enzyme replenishment. The cumulative engineering and identification of hydrolases for PET degradation has resulted in the creation of a new PET hydrolase enzyme class (EC 3.1.1.101) in 2016.^[^ ^56^ ^]^

Commercialization of this new enzyme class is currently experiencing rapid growth as regulations on the recyclability of plastics continue to tighten, improving the economic viability of recycled plastics.^[^ ^60^ ^]^ Carbios, a French biotech company, is a notable leader in this field, demonstrating the production of PET bottles from 100% depolymerized PET. The company is preparing for its first commercial plant in 2025.^[^ ^56^ ^]^ Other innovators in the space include Samsara Eco (Australia), Epoch Biodesign (United Kingdom), and Birch Biosciences (United States), which are at various stages of developing commercial processes and facilities.^[^ ^61^ ^]^ Should readers wish to read more in‐depth about the enzymatic degradation of PET, a recently published paper by Tournier et al. provides an excellent summary of the field.^[^ ^56^ ^]^ Further information on the depolymerization and upcycling of PET can also be found in other references.^[^ ^59^, ^62^, ^63^, ^64^ ^]^ Overall, the successful enzymatic depolymerization of PET and the commercialization of this process demonstrate that there may be a viable path forward for other plastics, and suitable depolymerization or degradation pathways are likely to be discovered. The research on the degradation of other plastics can greatly benefit from the lessons learned as PET depolymerization is commercialized and matured.

#### 4.1.2 Enzymatic degradation of PE

PE poses a considerable challenge to enzymatic degradation. PE is the most produced plastic at ∼103 million metric tons per year (∼27% of plastics) and comes in a variety of types, characterized by their branching morphology and molecular weight.^[^ ^57^ ^]^ The main varieties of PE are high‐density (52%), low‐density (18%), and linear‐low‐density (30%) PE, with niche varieties including high‐molecular‐weight and cross‐linked PE. The low surface energy and lack of heteroatoms in PE necessitate the introduction of oxygen as the first step for degradation. However, this is kinetically unfavorable, especially within the crystalline domains of PE.^[^ ^65^ ^]^ Therefore, varieties such as high‐density, cross‐linked, and high‐molecular‐weight PE are usually avoided when testing enzymes because of their high crystallinity, poor access to individual chains, and reduced number of tertiary carbons. Instead, low‐density (LDPE) and low‐molecular‐weight (LMWPE) varieties are favored, although they are not representative of most commercial high‐density polyethylene (HDPE).

**FIGURE 2:**
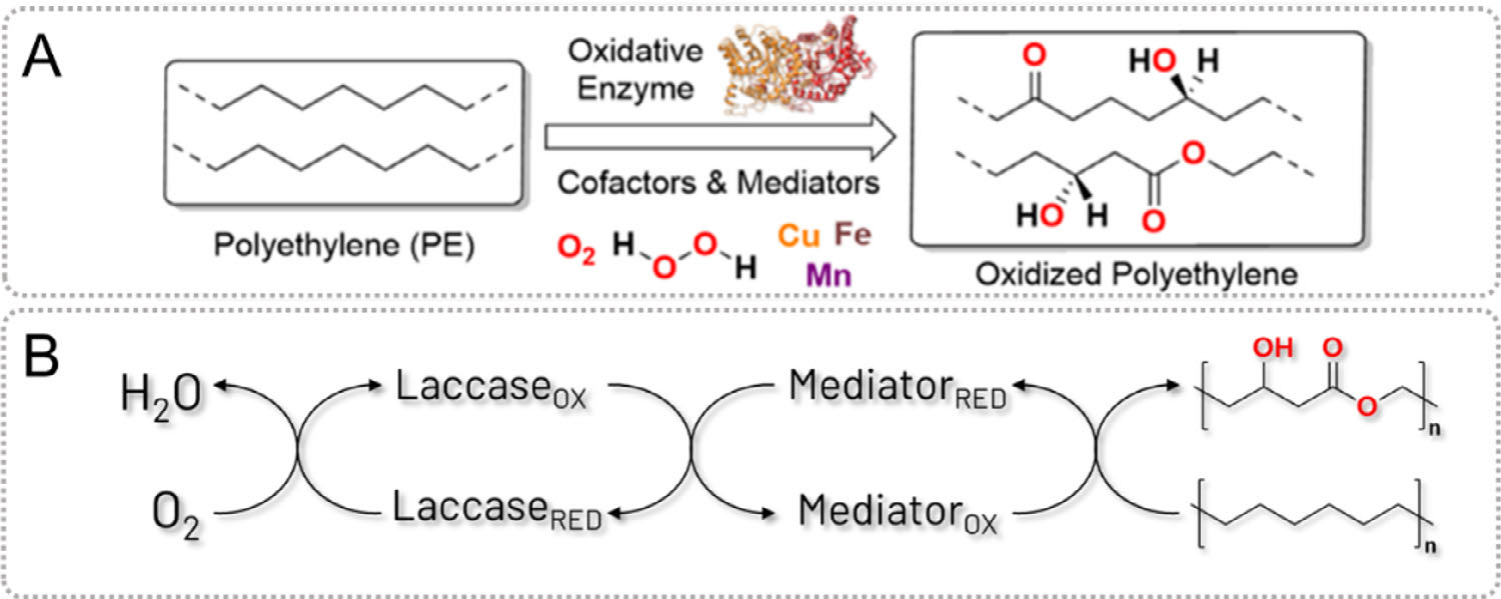
(A) General scheme for the study of oxidative enzymes for polyethylene (PE) degradation depicting common cofactors, mediators, and PE structure before and after degradation. (B) General scheme of the laccase mediator system (LMS) used to oxidize PE.

Enzymes previously reported to be involved in the degradation of PE include peroxidases, laccases, alkane hydroxylases, and phenol oxidases (Figure 2A).^[^ ^66^, ^67^, ^68^, ^69^, ^70^, ^71^, ^72^, ^73^, ^74^, ^75^, ^76^, ^77^ ^]^ A summary of the enzymes reported to degrade PE and the subsequent analytical methods is presented in Table 1. Manganese peroxidase (MnP) was the first purified enzyme reported to degrade PE as determined by reductions in tensile strength and weight average molecular weight (from 716,00 to 89,500 after 8 days).^[^ ^66^, ^67^ ^]^ A study attempted to repeat this success with MnP but saw limited changes to either the PE weight or FTIR spectrum even with a UV pretreatment.^[^ ^74^ ^]^ Soybean peroxidase was another early avenue of investigation with indications of oxidation by XPS, water contact angle, SEM, and FTIR.^[^ ^69^ ^]^ However, the use of hydroquinone in the treatment solution may result in entrapment in the PE, potentially confounding nonspecific analysis methods such as XPS, FTIR, and SEM. The short incubation period of only 15 min also raises some doubts regarding the efficacy of oxidation. However, additional studies with peroxidases are also warranted, but careful control of the PE substrate, consideration of any mediators used, and a more comprehensive chemical analysis are necessary.

**TABLE 1:** Literature review of purified enzymes used for polyethylene degradation.

Type of PE	Type of enzyme	Enzyme class	Source	Analysis methods	Observations	Reference
High‐molecular‐weight	Manganese peroxidase	EC 1.11.1.13	Isolated from *Phanerochaete chrysosporium*	GPC, relative elongation, relative tensile strength	Chain cleavage and reduction in integrity	[66, 67]
Unspecified membrane	Laccase	EC 1.10.3.2	Isolated from *Tinea versicolor*	GPC, relative elongation, relative tensile strength	Chain cleavage and reduction in integrity	[69]
High‐density	Soybean peroxidase	EC 1.11.1.7	Shang Hai Bio‐chemical Co.	XPS, FTIR, water contact angle, SEM, UV‐Vis (methyl violet stain)	Introduced oxygen, increased surface roughness and hydrophilicity	[68]
UV‐pretreated Low‐density	Laccase	EC 1.10.3.2	Isolated from *Rhodococcus ruber*	GPC, FTIR‐ATR, weight loss, DSC	Crystallinity increased and chain cleavage	[70]
UV‐pretreated Low‐density	Laccase	EC 1.10.3.2	Isolated from *T. versicolor*	GPC, FTIR‐ATR, weight loss, DSC	No effect	[70]
Low‐molecular‐weight	Alkane hydroxylase	EC 1.14.15.3	Isolated from *Pseudomonas sp*.	CO_2_ emission, SEM	Never isolated, increased CO_2_ emission with PE, SEM roughness	[71, 72, 73]
UV‐pretreated Low‐density	Manganese peroxidase	EC 1.11.1.13	Isolated from *Trichoderma harzianum*	Weight loss, FTIR	0.6% weight loss, limited changes to FTIR	[74]
UV‐pretreated Low‐density	Laccase	EC 1.10.3.2	Isolated from *T*. *harzianum*	Weight loss, FTIR	0.5% weight loss, limited changes to FTIR	[74]
Low‐molecular‐weight/Unspecified film	Phenol oxidase/Hemocyanin (Demetra)	EC 1.10.3.2	Isolated from *Galleria mellonella*	Raman, GPC, FTIR, SEM, GC‐MS	Introduced oxygen, limited chain cleavage, surface roughness, oxidized by‐products	[75]
Low‐molecular‐weight/Unspecified film	Phenol oxidase/Hemocyanin (Ceres)	EC 1.10.3.2	Isolated from *G. mellonella*	Raman, GPC, FTIR, SEM, GC‐MS	Introduced oxygen, limited chain cleavage, no visible change, no byproducts detected	[75]
UV‐pretreated Low‐density	Laccase	EC 1.10.3.2	Isolated from *Botrytis aclada*	SEM, FTIR‐ATR, GPC, GC‐MS	Surface roughness, increased oxygen inclusion, chain cleavage, metabolites detected	[76]
UV‐pretreated Low‐density	Laccase	EC 1.10.3.2	Isolated from *Bacillus subtilis*	SEM, FTIR‐ATR, GPC, GC‐MS	Surface roughness, increased oxygen inclusion, chain cleavage, metabolites detected	[76]
Unspecified film and powder	Laccase	EC 1.10.3.2	Isolated from *Psychorobacter sp*. NJ228	SEM, water contact angle, XRD, weight loss	Surface roughness, decreased water contact angle, decreased crystallinity, 13.2% weight loss	[77]

Abbreviations: DSC, differential scanning calorimetry; GPC, gel permeation chromatography; GC‐MS, gas chromatography‐mass spectrometry; FTIR, Fourier transform infrared; PE, polyethylene; SEM, scanning electron microscopy; XPS, X‐ray photoelectron spectroscopy; XRD, X‐ray diffraction.

Most PE degradation studies have focused on laccases, particularly the laccase‐mediator system (LMS), as shown in Figure 2B. A major advantage of LMS is the use of molecular oxygen as an electron acceptor instead of hydrogen peroxide, eliminating the need for constant addition of the oxidizer. One of the earliest reports of the LMS for the degradation of PE utilized 1‐hydroxybenzotriazole (HBT) as a radical mediator, though 2,2′‐azino‐bis(3‐ethylbenzothiazoline‐6‐sulfonic acid) (ABTS) and (2,2,6,6‐tetramethyl‐1‐piperidinyl)oxidanyl (TEMPO) have also been used with varying success in subsequent studies, and in some cases, laccase was used without a mediator.^[^ ^69^, ^70^, ^76^, ^77^ ^]^ There is some disagreement regarding whether pretreatment with UV irradiation is necessary for activity. Additional disagreement also exists as to the efficacy of laccase sourced from *Trametes versicolor*.^[^ ^69^, ^70^ ^]^ However, laccase is currently the most well‐developed and well‐understood enzyme among those suggested to modify PE, likely representing a future path forward for research.

Innovative alternatives to laccases and peroxidases for PE degradation have also been explored using a variety of approaches in recent years. Notably, an extensive analysis of *Pseudomonas aeruginosa* sourced from oil‐degrading bacteria was completed in South Korea.^[^ ^71^, ^72^, ^73^ ^]^ From this analysis, a pair of alkane hydroxylases was identified as being responsible for LMWPE mineralization; however, the responsible enzymes were not isolated. Additionally, a separate study explored wax worm saliva as a potential source of PE‐degrading enzymes.^[^ ^75^ ^]^ This exploration rationally originates from the fact that bees wax, the typical food of wax worms, shares some chemical similarities with PE and the observation that they will chew plastic bags.^[^ ^75^ ^]^ However, upon analysis, negligible decreases to the molecular weight and limited signs of oxidation even after exposure to high enzyme concentrations have created doubt within the community about the enzymatic efficiency of the two enzymes isolated.^[^ ^56^ ^]^ Nevertheless, novel studies, such as these, with environmental observations as their basis for enzymatic experimentation are the most likely to yield revelations for the field.

#### 4.1.3 Enzymatic degradation of PS

Polystyrene (PS) is an amorphous vinylic polymer that is produced at a global annual rate of ∼19.8 million metric tons per year.^[^ ^57^ ^]^ Though it represents about 5% of all plastic produced, only a single experiment has examined the degradative ability of purified enzymes for PS.^[^ ^78^ ^]^ This study used hydroquinone peroxidase sourced from *Azotobacter beijerinckii*, along with hydrogen peroxide and tetramethylhydroquinone, to degrade high‐molecular‐weight PS. Degradation of PS occurred in a two‐phase solvent system (dichloromethane‐water), with PS dissolved in the organic phase and the enzyme system dissolved in the aqueous phase, and degradation occurred at the solvent interface. Degradation products were confirmed by HPLC, yielding a molecular weight of ∼350 after only 10 min of treatment. The peroxidase did not degrade PE, poly(methyl methacrylate) (PMMA), PP, or polyethylene glycol (PEG), suggesting that the aromaticity in the PS benzyl rings is potentially responsible for the lability. While the results from this study were very promising, further research is needed to characterize this degradation pathway.

Though research on PS degradation with purified enzymes is lacking, a number of biodegradation studies indirectly point to the possible role of active enzymes.^[^ ^64^, ^65^, ^78^, ^79^, ^80^, ^81^, ^82^, ^83^ ^]^ Recently, data mining of these studies has revealed the most likely enzymes to be cytochrome P450s, monooxygenases, and aromatic ring hydroxylases.^[^ ^83^ ^]^ The main bacteria phyla that are suspected to produce PS‐degrading enzymes are *Proteobacteria*, *Actinobacteria*, *Bacterioidota*, and *Firmicutes*.^[^ ^83^ ^]^ An excellent source for additional reading on the biodegradation of PS was published by Guo et al.^[^ ^84^ ^]^ In general, enzymatic PS degradation has been relatively unexplored compared to that of other polymers. However, the similarities between PS and natural polymers, such as lignin, suggest a high potential for enzymatic degradation.

### 4.2 Degradation based on Fenton chemistry

#### 4.2.1 Reaction mechanism

Fenton oxidation, which relies on the generation of highly reactive hydroxyl radicals (•OH) through the reaction of iron catalysts and hydrogen peroxide (H_2_O_2_), is a promising approach for microplastic degradation.^[^ ^85^ ^]^ Fenton chemistry applied to microplastic degradation involves a sequence of reactions. Initially, the iron catalyst, typically ferrous iron (Fe^2+^), reacts with hydrogen peroxide, leading to the formation of ferric ions (Fe^3+^) and hydroxyl radicals via the Fenton reaction:

$${\mathrm{Fe}}^{2+}+H_{2}O_{2}\to {\mathrm{Fe}}^{3+}+•\mathrm{OH}+{\mathrm{OH}}^{-}$$

$${\mathrm{Fe}}^{3+}+H_{2}O_{2}\to {\mathrm{Fe}}^{2+}+•\mathrm{OOH}+H^{+}$$

**FIGURE 3:**
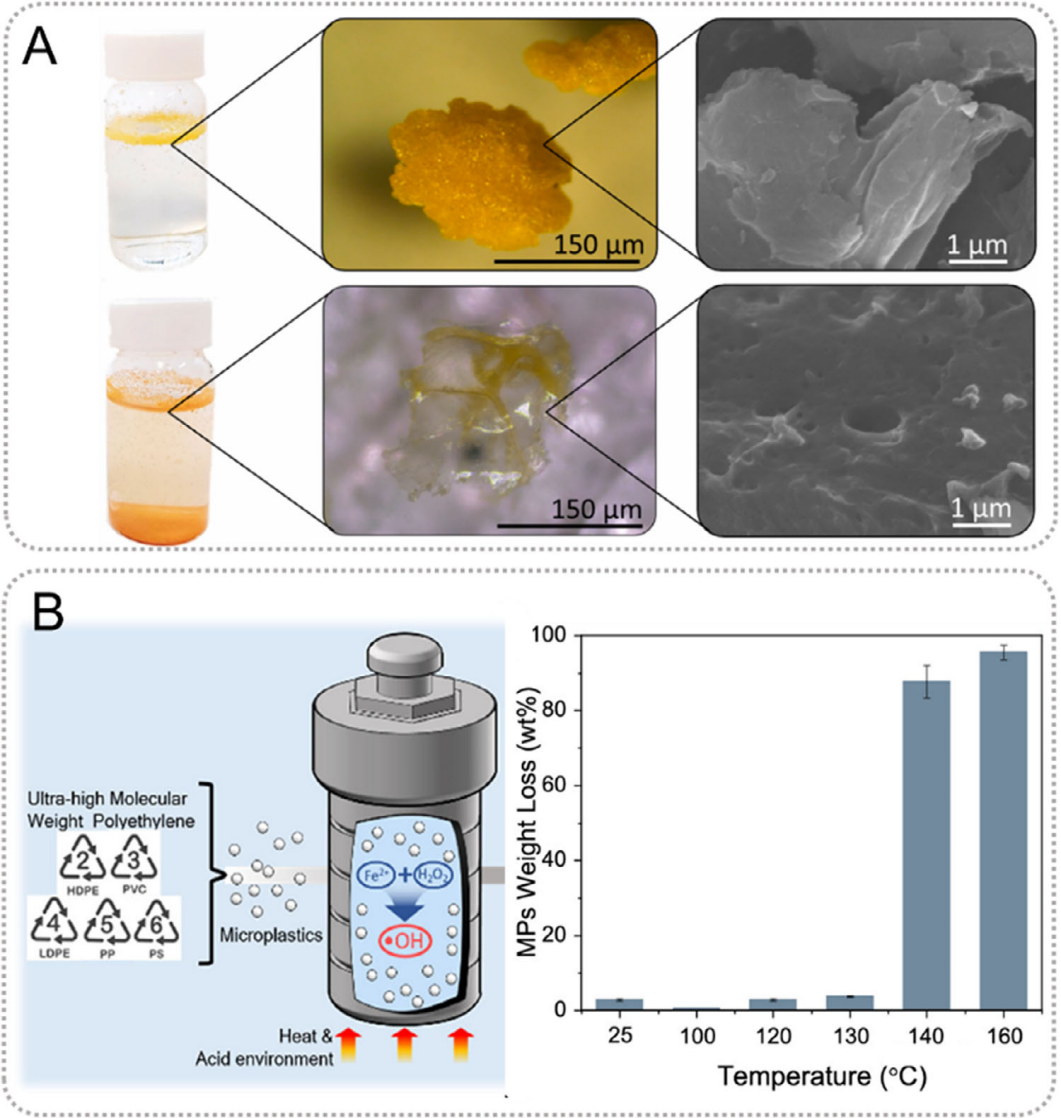
(A) Comparison of suspensions, light microscopy images, and scanning electron microscopy (SEM) images of fresh (top row) and degraded (bottom row) expanded polystyrene (EPS) microplastics. Reproduced under the terms of the CC‐BY Creative Commons Attribution 4.0 International license (https://creativecommons.org/licenses/by/4.0).^[^ ^86^ ^]^ Copyright 2022, The Authors, published by Elsevier. (B) Effect of elevated temperature on Fenton‐assisted degradation and weight loss of polyethylene (PE) microplastics. A schematic of the microplastic degradation process is depicted in the left panel. At temperatures above 140°C, a remarkably high degree of degradation was achieved. Reproduced with permission.^[^ ^91^ ^]^ Copyright 2022, American Chemical Society.

These hydroxyl radicals then attack the polymer chains of microplastics, leading to the breakdown of the polymer structure into smaller fragments. The subsequent reactions involve the breaking of polymer bonds, formation of oxygenated functional groups, and mineralization of the degraded microplastic fragments, ultimately producing simpler and less toxic by‐products. The degradation of microplastics by Fenton oxidation has been thoroughly evaluated based on the weight loss (Figure 3A).^[^ ^86^ ^]^ In this study, five types of microplastic particles, including PET, PE, PVC, PP, and expanded polystyrene (EPS) in the size range of 150−250 μm, were digested for 7.5 h under relatively severe operational conditions of 80°C, pH_0_  =  3, [H_2_O_2_]_0_  =  1000 mgL^−1^ administered in 15 doses, one every 0.5 h; [Fe^3+^]_0_  =  10 mgL^−1^ (5 doses, 1 every 1.5 h).

In the context of cleaning microplastics for analysis, the Fenton reaction is used to remove adhered or absorbed contaminants from the surface of microplastics. When the Fenton reaction is used to degrade microplastics themselves, the conditions typically involve the use of higher concentrations of hydrogen peroxide and iron ions to break down the microplastic polymers themselves.

#### 4.2.2 Combining Fenton chemistry with other methods

The combination of Fenton reactions with other techniques such as ultrasound, electrochemistry, photocatalysis, and thermal treatment can result in synergistic effects, enhancing the degradation efficiency and broadening the scope of applications. For example, exposure to ultrasound has been reported to enhance the degradation of organic pollutants^[^ ^87^ ^]^ and reduce additive leaching from microplastics.^[^ ^88^ ^]^ Electrochemical methods are particularly effective for PVC microplastic degradation.^[^ ^89^, ^90^ ^]^ Additionally, thermal treatment was explored for microplastic degradation, since elevated temperatures and melting of plastic particles facilitate the access of reactive oxygen species to polymer chains (Figure 3B).^[^ ^91^ ^]^ By combining these processes with complementary techniques, it is possible to maximize the efficiency and effectiveness of microplastic degradation. These synergistic effects may provide new opportunities for improved remediation strategies and contribute to addressing the challenges posed by microplastic pollution.

#### 4.2.3 Advantages and challenges

Fenton remediation has been shown to have a high effectiveness‐to‐cost ratio making it an attractive option for microplastic remediation.^[^ ^92^ ^]^ Fenton and Fenton‐like systems rely on the in situ production of highly reactive radical species. One key advantage of the Fenton process is its versatility and applicability to various types of microplastics found in diverse environmental matrices such as water bodies, soils, and sediments encompassing different polymer materials and sizes. Fenton remediation offers additional advantages such as easy catalyst regeneration and environmental compatibility. In addition, the products resulting from microplastic degradation through Fenton oxidation can serve as growth and energy substrates for microorganisms.^[^ ^93^ ^]^

Despite the potential use of Fenton chemistry for microplastic degradation, several challenges need to be addressed. The first challenge is the precipitation of Fe^3+^ solid phases, which occurs when the catalyst (Fe^2+^) oxidizes, hence causing the loss of the catalyst as well as changes in pH. To overcome this limitation, Fenton‐like reactions^[^ ^94^ ^]^ have emerged as alternatives to microplastic degradation. Some other challenges include optimizing various parameters, such as the type and concentration of the iron catalyst, hydrogen peroxide concentration, pH, temperature, and reaction time. The variable characteristics of microplastics and environmental matrices affect the efficiency of Fenton remediation. Selective degradation of targeted plastics, while minimizing their impact on non‐targeted materials, is especially challenging. The degradation rates by Fenton reactions can be low for large or complex plastics. Enhancing the efficiency, stability, and reusability of Fe catalysts is crucial for the economic feasibility of Fenton‐based remediation. Advanced iron catalysts, such as zero‐valent iron nanoparticles^[^ ^90^ ^]^ and iron‐based composites,^[^ ^95^ ^]^ may offer improved performance.

### 4.3 Degradation by nanomaterials

For catalysis, nanomaterials provide a larger surface‐to‐volume ratio, thereby increasing the number of catalytically active surface sites. In addition, nanoscale size facilitates the accessibility of catalysts to confined environments, such as cells in biomedical research or subsurface environmental systems. Utilizing nanomaterials for the degradation of microplastics can generally fall into the following broad categories.

#### 4.3.1 Photocatalytic nanomaterials

**FIGURE 4:**
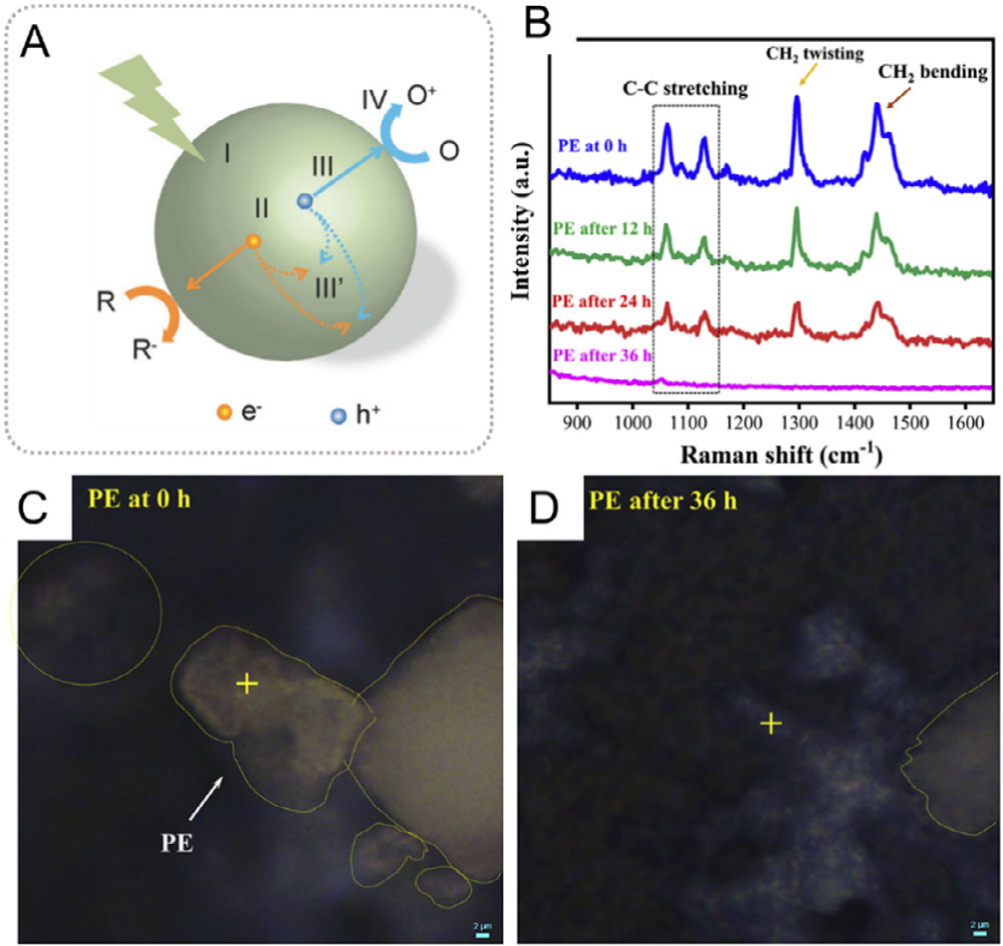
(A) Photocatalytic reaction mechanism of materials: (I) light absorption, (II) separation of excited charges, (III) transfer of electrons and holes to the surface, and (IV) charge‐induced redox reaction on the surface. Reproduced with permission.^[^ ^97^ ^]^ Copyright 2017, Wiley‐VCH. Photocatalytic degradation of polyethylene (PE) plastics induced by 254‐nm UV light irradiation on TiO_2_: (B) Raman spectra of the PE at different degradation time intervals. (C,D) Raman images of PE samples degraded over 36 h. Reproduced under the terms of the CC‐BY Creative Commons Attribution 4.0 International license (https://creativecommons.org/licenses/by/4.0).^[^ ^104^ ^]^ Copyright 2020, The Authors, published by Elsevier.

The decomposition and degradation of microplastics can be achieved by UV irradiation on photocatalytic nanomaterials. Upon UV irradiation (e.g., on TiO_2_), photoexcited electron–hole pairs are generated, and their migration induces a redox reaction at the surface of nanomaterials (Figure 4A).^[^ ^96^, ^97^ ^]^ To facilitate the access of light and photocatalysts to the polymer chains, pretreatment steps are normally conducted. Dissolving plastics in organic solvents such as tetrahydrofuran^[^ ^98^ ^]^ or cyclohexane^[^ ^99^, ^100^, ^101^ ^]^ is a common method that allows for homogenous incorporation of photocatalysts or other modifiers on the plastic surface to enhance the degradation efficiency. Moreover, surface modification of photocatalytic nanomaterials to modulate the surface electronic structure can facilitate the upcycling of plastic and microplastic wastes to be transformed into high‐value products such as C_2_ fuels,^[^ ^102^ ^]^ methane, and acetic acid.^[^ ^103^ ^]^

TiO_2_ nanoparticles are the most common photocatalysts used for plastic and microplastic degradation, and various plastic materials, including PE, PS, and PVC have been degraded using it (Figure 4B–D). Typically, a nanocomposite of TiO_2_ nanoparticles adsorbed onto a plastic film is used to achieve photocatalytic degradation. Depending on the composing material of the microplastics, concentration of TiO_2_ nanoparticles, irradiation source, and duration of irradiation, various degradation efficiencies in the form of weight loss (mostly greater than 20%) were achieved (Table 2). In contrast, control experiments showed that in the absence of TiO_2_ nanoparticles, a weight loss of less than 1% was achieved under the same light irradiation.^[^ ^104^ ^]^

**TABLE 2:** Literature review of photocatalytic degradation of various plastic materials achieved by light irradiation on TiO_2_ nanoparticles.

Plastics	[TiO_2_]	Light source	Irradiation duration (h)	Weight loss (%)	Reference
PVC film	1.5 wt%	UV (300 nm < λ < 400 nm)	300	27	[98]
LDPE film	2 wt %	solar	250	26	[105]
LDPE film	1 wt%	solar	300	42	[99]
LDPE film	0.1 wt%	UV (15W@ 365 nm)	300	18	[100]
LDPE film	0.1 wt%	solar	200	68	[101]
LDPE film	10 wt%	UV (36W@ 315 nm)	360	78	[106]
PS microspheres	Nanofilm	UV (256W@ 254 nm)	12	98	[104]
PE microspheres	Nanofilm	UV (256W@ 254 nm)	36	100	[104]

Abbreviations: PVC, polyvinyl chloride; PE, polyethylene; LDPE, low‐density polyethylene.

Other nanomaterials such as ZnO, MnO_2_, Cu_2_O, and CdS have also shown photocatalytic activity. Moreover, the conjugation of more than one nanomaterial can enhance the photocatalytic activity for microplastic degradation. The results of some of these studies are summarized in Table 3. As a representative example, Uekert et al. used hybrid CdS/CdO_x_ quantum dots in an alkaline environment under solar irradiation to degrade various plastic materials and generate H_2_ and organic compounds such as formate, pyruvate, and acetate.^[^ ^107^ ^]^ Graphene oxide (GO) is another nanomaterial that can facilitate the transportation and sustainability of photogenerated electrons. Therefore, conjugation of GO with other photocatalysts, such as metal oxides, not only enhances the photocatalytic activity but also helps with binding efficiency with substrates.^[^ ^108^ ^]^ Fadli et al. designed a hybrid nanomaterial consisting of Ag, TiO_2_, and rGO, which resulted in a ∼20% higher weight loss of PE microplastics upon UV irradiation compared to the absence of rGO.^[^ ^109^ ^]^

**TABLE 3:** Literature review of photocatalytic degradation of various plastic materials achieved by light irradiation on various nanoparticles.

Plastics	Material	Light source	Irradiation duration (h)	Weight loss (%)	Reference
PVC film	ZnO	UV (22W@365 nm)	2	20	[110]
PP microspheres	ZnO	Visible (120 W)	456	65 (volume loss)	[111]
PE film	TiO_2_/MnO_2_	UV (6W@280 nm)	90	21	[112]
PE microplastics	GO	UV (72W@350 nm)	8	36	[108]
PE microplastics	GO/TiO_2_	UV (72W@350 nm)	8	50	[108]
PE microplastics	GO/MnO_2_	UV (72W@350 nm)	8	40	[108]
PE microplastics	GO/Cu_2_O	UV (72W@350 nm)	8	48	[108]

Abbreviations: PVC, polyvinyl chloride; PE, polyethylene; PP, polypropylene.

#### 4.3.2 Nanozymes

**FIGURE 5:**
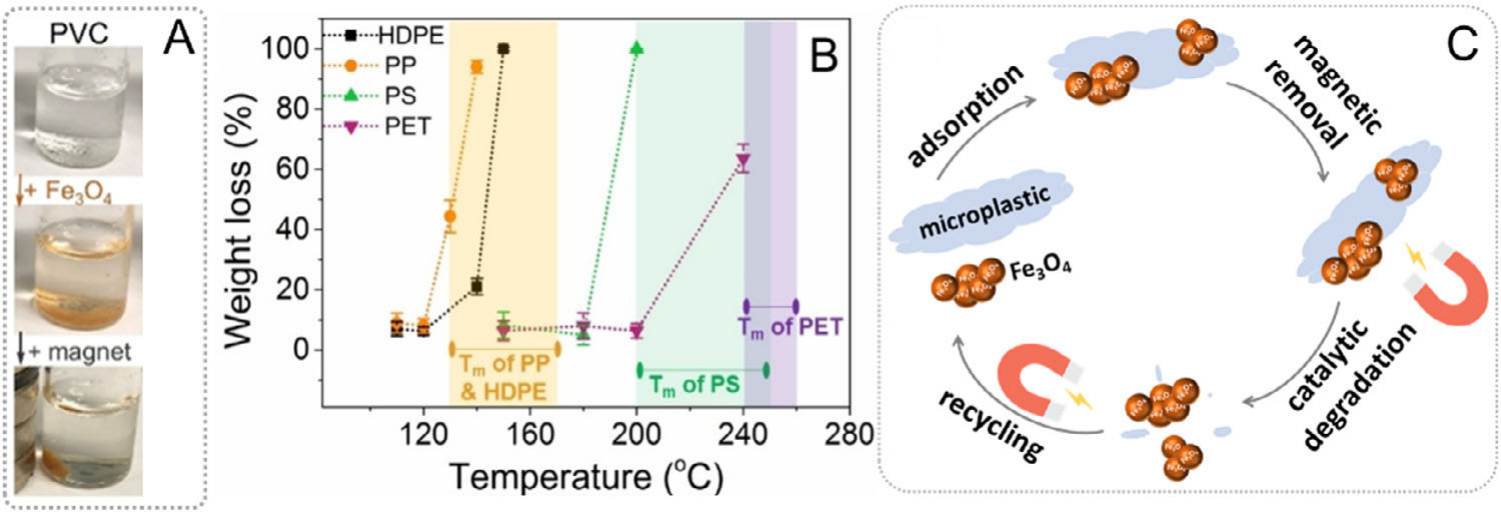
(A) Photographs illustrating the adsorption of bare Fe_3_O_4_ nanoparticles onto polyvinyl chloride (PVC) microplastics and the magnetic removal of microplastics. (B) Catalytic degradation by bare Fe_3_O_4_ nanoparticles and weight loss of various microplastics materials heated at different temperatures. Each microplastic material was efficiently degraded at temperatures close to its melting point. (C) A scheme depicting the cycle of Fe_3_O_4_ nanoparticles for the removal and catalytic degradation of microplastics. Reproduced with permission.^[^ ^23^ ^]^ Copyright 2022, Wiley‐VCH.

Nanozymes are catalytic nanomaterials that have identical substrates and products as enzymes.^[^ ^113^ ^]^ Fe_3_O_4_ is among the most studied nanozymes with peroxidase‐like activities.^[^ ^114^ ^]^ Our team has recently investigated the adsorption of bare Fe_3_O_4_ nanoparticles onto the most abundant microplastic materials to achieve magnetic removal (Figure 5A).^[^ ^23^ ^]^ While melting microplastics facilitates access to polymer chains, natural enzymes cannot survive at such high temperatures. On the contrary, one of the attractive properties of nanozymes is their high stability against harsh conditions, and they are even more active at elevated temperatures.^[^ ^115^ ^]^ Therefore, the Fe_3_O_4_ nanozyme induced catalytic degradation of microplastics at temperatures close to the melting point of the microplastics, and nearly 100% weight loss was achieved using this method (Figure 5B). Moreover, the Fe_3_O_4_ nanoparticles could be recovered for subsequent degradation of the microplastics (Figure 5C). In another study, Knag et al. used manganese‐coated carbon nanosprings for catalytic activation of peroxymonosulfate to generate reactive radicals for decomposing commercial cosmetic plastic microbeads.^[^ ^116^ ^]^ They observed the temperature‐dependant degradation efficiency, and at a temperature of 160°C, 54% weight loss of microplastics was achieved. Palliyarayil et al. have recently emphasized the potential of nanozymes and artificial enzymes for degrading microplastics.^[^ ^117^ ^]^

#### 4.3.3 Enzyme‐immobilized nanomaterials

Another type of nanozyme is prepared by immobilization (either physical adsorption or covalent binding) of enzymes/microorganisms on nanomaterials, which can be useful for microplastics degradation for various reasons. (1) Some nanomaterials are catalytically active intrinsically, thereby providing a hybrid catalyst.^[^ ^118^ ^]^ (2) Nanomaterials can provide a wide range of functionalities other than catalysis, such as magnetic separation provided by Fe_3_O_4_ nanoparticles,^[^ ^119^, ^120^ ^]^ or high adsorption efficiency provided by polydopamine.^[^ ^121^ ^]^ (3) Immobilized enzymes can be protected by nanomaterials improving the thermal stability of enzymes.^[^ ^122^, ^123^ ^]^ (4) In some cases, nanomaterials aided bacterial growth used for biodegradation.^[^ ^124^, ^125^ ^]^

In a representative example, Li et al. immobilized the DuraPETase enzyme on Fe_3_O_4_ nanoparticles to investigate solar‐driven enzymatic degradation of PET.^[^ ^126^ ^]^ Fe_3_O_4_ nanoparticles could elevate the reaction temperature from 25°C to 46°C upon solar irradiation, which boosted the degradation efficiency sixfold compared to the absence of Fe_3_O_4_ nanoparticles. Interestingly, they indicated that although immobilization on the nanoparticles increased the stability of the enzyme when the enzyme and Fe_3_O_4_ nanoparticles were added separately (no immobilization), the catalytic degradation efficiency was even higher than that of the immobilized system.

In another study, Zhou et al. immobilized lipase on polydopamine‐coated Fe_3_O_4_ nanoparticles to enhance enzymatic activity for the degradation of polycaprolactone microplastics.^[^ ^121^ ^]^ The magnetic removal of microplastics was achieved owing to the Fe_3_O_4_ core, and the adhesiveness of polydopamine facilitated the proximity of lipase to microplastics for efficient degradation performance. Species other than enzymes can also be conjugated with nanomaterials to upcycle microplastics. Ye et al. integrated *Methanosarcina barkeri* with carbon dot‐functionalized polymeric carbon nitrides (as a photocatalytic nanomaterial) for biodegradation of polylactic acid microplastics.^[^ ^127^ ^]^

### 4.4 Microbe‐assisted degradation

Microbe‐assisted degradation refers to the breakdown of polymers by environmental microbes, such as those found in soil and water, or those genetically modified in the laboratory.^[^ ^128^ ^]^ The majority of polymer degradation research has been conducted in laboratory settings with modified organisms or single isolates. An important motivation for this research is to design environmentally friendly bioremediation methods for plastic waste. Controlled laboratory experiments, however, face challenges when scaling up to real‐world environmental applications.^[^ ^129^ ^]^ Because much work has focused on soil and landfill microorganisms in laboratory settings, the focus of this section will be on the microbe‐assisted degradation of microplastics in freshwater and marine conditions.

**FIGURE 6:**
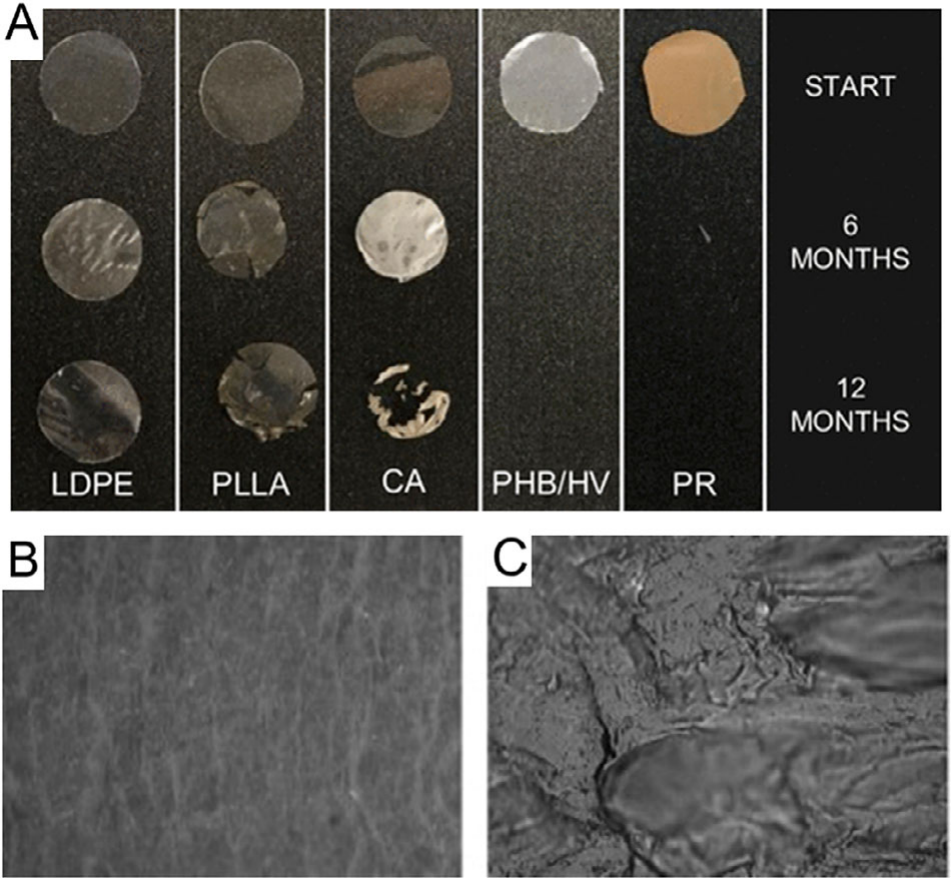
(A) A comparison of bioplastic and low‐density polyethylene (LDPE) plastic degradation rates over 1 year in the Baltic Sea revealed visual degradation. Reproduced under the terms of the CC‐BY Creative Commons Attribution 4.0 International license (https://creativecommons.org/licenses/by/4.0).^[^ ^146^ ^]^ Copyright 2022, The Authors, published by American Chemical Society. Scanning electron microscopy (SEM) micrograph showing the surface alteration of a nylon 66 pellet (B) before and (C) after 3 months of exposure to *Bacillus cereus*. Reproduced with permission.^[^ ^138^ ^]^ Copyright 2022, Wiley‐VCH.

Complete biodegradation of LDPE and HDPE has not been achieved (see Figure 6A for degradation of LDPE over a year in situ),^[^ ^130^ ^]^ which explains the relatively high accumulation of PE waste seen in the environment.^[^ ^131^ ^]^ This has prompted research in pretreatments of PE that enable microbial degradation. Common pre‐treatments include exposure to UV irradiation, as well as thermo‐oxidation. Both pre‐treatments can break polymer carbon chains giving microorganisms access points to begin degradation. Some microorganisms may even create their access points for degradation via hydroperoxidation.^[^ ^132^ ^]^ The potential toxicity of PE biodegradation, including the effect of toxic by‐products, has not been determined to date.^[^ ^133^ ^]^

Table 4 presents a partial list of studies conducted in marine and freshwater environments that have investigated (micro)plastic degradation by microorganisms. A more recent study extracted 248 bacterial isolates from a plastic waste dumping site in Tamil Nadu, India, and examined their ability to degrade HDPE. The results confirmed that most of the degrading isolates belonged to the genus *Bacillus* spp., which are common marine and freshwater bacteria (see Figure 6B,C for degradation caused by *Bacillus cereus*), as well as *Pseudomonas* spp.​^[^ ^140^ ^]^ In another study, *Alcanivorax borkumensis* was observed to form a biofilm on LDPE and attack petroleum‐based polymers.^[^ ^141^ ^]^ Interestingly, the bacterium naturally propagates and is found in seawater containing crude oil when nutrient nitrogen and phosphorus are available.^[^ ^142^ ^]^ These results suggest that this bacterium could be a potential candidate for bioremediation of (micro)plastic waste in marine environments.

**TABLE 4:** Literature review of catalytic degradation of (micro)plastics by microorganisms.

Plastic‐type	Conditions	Duration	Microorganisms detected/used	Weight loss	Other analyses	Reference
LDPE HDPE	Marine organisms using treated and untreated plastic, incubated at 30°C, pH 7.5	1 year	*Bacillus sphericus GC subgroup IV* *Bacillus cereus subgroup* A	***B. sphericus*** **LDPE** Untreated 10% Heat treated 24% **HDPE** Untreated 3.5% Heat treated 9.5% ***B. cereus*** **LDPE** Untreated 5% Heat treated 15% **HDPE** Untreated 2.1% Heat treated 7%	Decreased tensile strength and crystallinity Double bond changes using FTIR	[134]
LDPE	Isolated 60 marine bacteria from pelagic water (Arabian Sea) Incubation with ZMB	30 days' incubation, temperature unknown	*Kocuria palustris* M16 *Bacillus pumilus* M27 *Bacillus subtilis* H1584.	***K. palustris*** 1% ***B. pumilus*** 1.5% ***B. subtilis*** 1.75%	FTIR changes to keto carbonyl and ester carbonyl bonds	[135]
LDPE powder	Using fungi isolated from seawater (origin Bay of Bengal) Yeast presence at room temperature	10 days	*Aspergillus spp*.	N/A	SEM showed evidence of biodegradation	[136]
E(PS)	Bacteria originating from estuarine areas in Zi‐No Town, China Collected ESP environmental samples and identified bacteria present Bacteria enriched, temperature 28°C with MMC	30 days	***Gordonia*** **strain** *ZN14R1* *ZN15R9* *ZN17RX* ***Novosphingobium strain*** *ZN18A2*	***Gordonia*** **strain** *ZN14R1*, 4.69% *ZN15R9*, 7.73% *ZN17RX*, 6.69% ***Novosphingobium strain*** *ZN18A2*, 2.66%	FTIR changes	[137]
PA 6 and 66	Artificial seawater at 35°C Using marine bacteria from the Indian Ocean	3 months	*B. cereus* *Brevundimonas vesicularis*	***B. cereus*** *PA 66*, 7% *PA 6*, 2% ***B. vesicularis*** *PA 66*, 4.5% *PA 6*, 2%	N/A	[138]
PET	Marine bacteria collected from plastic debris samples from the Bay of China	2 weeks	*Exiguobacterium* sp. *Halomonas* sp. *Ochrobactrum* sp.	N/A	Degradation visible through SEM and FTIR Macro transcriptome analysis to confirm polymer was the energy source	[139]

Abbreviations: FTIR, Fourier transform infrared spectroscopy; HDPE, high‐density polyethylene; LDPE, low‐density polyethylene; PET, polyethylene terephthalate; SEM, scanning electron microscopy.

An in situ study conducted in the North Sea investigated the variability in microbial species colonizing marine plastics during a 6‐week PET exposure experiment.^[^ ^143^ ^]^ SEM imaging showed that the thickness of the biofilm adhering to PET fragments increased from winter to summer and varied from one location to another. A previous study with PET bottles at a wastewater treatment plant also found colonization by several bacterial sequences of concern, specifically from the genus *Tenacibaculum*, which includes fish pathogens, as well as *Vibrio* and several other potential pathogens.^[145]^A study conducted in four nearshore marine locations in Japan, two urban areas, and two non‐urban areas analyzed the differences in degradation between sites. This study utilized polyamide powders and reported degradation rates dependent on location, with a weight loss of ∼70% at 6 weeks in the urban areas compared to about 10% at the bay entrance away from urban centers.^[^ ^145^ ^]^ The differences in biodegradation correlated with a significantly lower cell abundance on the microplastics in the non‐urban seawater.

The available research provides evidence that location, seasonality, and microbial community structure are important environment‐specific variables affecting microplastics degradation. Studies conducted in simplified laboratory settings may fail to consider multiple factors that affect the fate of microplastics in the environment. Hence, more in situ research should be conducted to fully characterize the degradation of microplastics in marine and freshwater ecosystems and to assess the associated ecological impacts. The presence of microplastics can alter microbial and faunal activity and disrupt the fragile ecosystem balance, whereas degradation products can be toxic. The implementation of remediation strategies to degrade environmental microplastics should carefully consider the possibility of unintentional negative consequences.

## 5 DEGRADATION IN THE ENVIRONMENT

A major concern regarding environmental microplastic degradation is the effect of breakdown products. Plastics, especially old and weathered plastics that are common in the environment, often leach into the surrounding areas upon environmental degradation. For instance, leaching from plastic debris in the oceans may cause acidification. According to some estimates, under business‐as‐usual scenarios, plastic pollution could cause a 0.5 pH unit decrease in surface seawater by 2100.^[^ ^147^ ^]^ Although this may seem like a small drop in pH, it could nonetheless have dire effects on marine life, especially calcifying organisms. With microplastic pollution in oceans and freshwater bodies increasing every year, significant disruption of ecosystem functions is expected. However, at present, the ecological impacts of microplastics pollution remain largely unknown. This emphasizes the importance of researching on the fate of microplastic degradation that can inform mitigation strategies and policies. The following are some of the major factors affecting the degradation of microplastics in environmental systems.

### 5.1 UV irradiation

When microplastic particles are exposed to sunlight, particularly UV‐B and UV‐A radiation, the energy from the UV photons can be absorbed by the plastic polymer chains, weakening the chemical bonds that hold the chains together. Specifically, the bonds most susceptible to UV‐induced cleavage are often carbon–carbon (C‐C) bonds. In addition to physical fragmentation, UV irradiation can induce the formation of new oxygen‐related functional groups on the microplastic surface altering the chemical and physical properties of the microplastics.^[^ ^148^ ^]^ Such photo‐assisted degradation may result in fragmentation and partial degradation of microplastics in the environment.^[^ ^149^, ^150^, ^151^ ^]^

### 5.2 Temperature, pH, and redox

Temperature, pH, and redox state are the major environmental variables affecting microplastic degradation. Increases in the temperature approaching the melting point of plastic polymers increase the degradation rate, which enhances the access of enzymes to polymer chains. However, the melting point of most plastics starts above 70°C,^[^ ^152^ ^]^ and very few natural enzymes survive or function at this temperature. Although some engineered enzymes have been shown to function better at higher temperatures,^[^ ^153^ ^]^ such temperatures are not relevant in most near‐surface environments where microplastics accumulate. Furthermore, most microorganisms in the environment have optimal temperatures around 30°C and, for the most part, tend to prefer near‐neutral pH conditions.^[^ ^154^, ^155^ ^]^ Microbial metabolism is also closely related to the prevailing redox conditions.^[^ ^156^ ^]^ Aerobic microorganisms are energetically favored over anaerobes that rely on electron acceptors other than molecular oxygen for their energy production, which, in turn, determines how much extracellular enzymes a cell can synthesize and release into its surroundings.^[^ ^157^ ^]^ Hence, microbially mediated microplastic degradation is likely to be increasingly limited by decreasing cellular energy yields as conditions become increasingly reduced. In addition, changing redox conditions in natural environments are accompanied by changes in pH, geochemical conditions, and biotic activity that can all alter the surface chemistry, physical properties, and accessibility of microplastics.^[^ ^154^, ^155^ ^]^

## 6 SUMMARY AND FUTURE PERSPECTIVES

In this article, we critically explored the current state of catalytic degradation of microplastics by reviewing a representative set of published studies. Various catalytic entities, including natural enzymes, microbes, metal ions (for Fenton chemistry), and nanomaterials, are being used to enable microplastic degradation, each with its advantages and shortcomings. On one hand, enzymes provide substrate selectivity while microbial consortia are beneficial for upcycling degradation products by biological processes. On the other hand, nanomaterials are robust and provide complementary functionalities, such as magnetic separation. Given the complexities and challenges of microplastics degradation, a combination of these approaches may hold the most promise. For example, enzyme‐inspired engineered nanomaterials (such as single‐atom nanozymes^[^ ^158^ ^]^) can combine the stability and functionality of nanoparticles with the selectivity of enzymes potentially offering a more versatile and efficient approach to microplastic degradation. However, it is essential to conduct rigorous research, consider the environmental and safety implications, and evaluate the efficiency and scalability of these technologies for specific applications and environmental contexts. A few key issues and perspectives regarding the catalytic degradation of microplastics are as follows.

- To begin with, the characterization of microplastics lacks standardization. As mentioned, the term “microplastic degradation” has been used to refer to a wide variety of outcomes, from the generation of oxygen‐containing functional groups on the surface to the complete mineralization of the organic polymer structure. In our opinion, various characterization methods, including spectroscopy, electron microscopy, weight loss measurements, and chromatography, should be viewed as complementary approaches for assessing the nature and extent of degradation. When a given characterization method is used, it should be clearly stated what variables are being measured and how they relate to the transformation of the microplastics in terms of changes in structural and functional properties and the generation of benign or high‐value products.
- The conjugation of catalytic entities and nanomaterials represents an interesting research avenue for microplastics degradation. Nanomaterials can add useful features to the reaction system to improve its stability, binding, separation, and activity. However, immobilization of enzymes on nanoparticles can compromise the enzyme activity itself because of the inactivation of catalytically active sites or disruption of enzyme–substrate binding sites. Conversely, increasing the enzyme stability may also yield a reactive system that is more sustainable for long‐term use. Therefore, the design of hybrid enzyme–nanomaterial conjugates should account for possible trade‐offs and anticipated end use.
- From a surface science viewpoint, the binding of different enzymes, nanozymes, and microorganisms to microplastics exerts a major influence on the degradation pathways and kinetics. For example, our team recently observed that upon soaking microplastics in water, the adsorption efficiency of biomolecules such as spherical DNA dramatically changed due to the change in the wettability of microplastics.^[^ ^159^ ^]^ Such adsorption studies should therefore be carried out systematically when assessing potential degradation catalysts for different microplastic substrates. These results can inform better experimental designs and, potentially, the engineering of more effective catalysts in terms of binding and activity.
- Scaling up and practical implementation of biotic, abiotic, and mixed catalytic degradation techniques are areas that require further investigation and development. Considering the diversity of bulk plastic waste in the waste stream, a universal degradation technique is favorable to target multiple plastic types effectively. Another important consideration for scaling up is sustainability, to ensure that the developed technique is economically and environmentally viable for large‐scale plastic waste treatment.
- Finally, the environmental degradation of plastic waste needs to be carefully evaluated with respect to the degradation products and multiple impacts on ecosystem health and services. Ideally, the end products of degradation pathways should be environmentally safe. To achieve such product selectivity, photocatalysis is a promising route, in which controlling the band structure of a photocatalyst may allow the activation of specific chemical bonds in a plastic structure. Moreover, the fields of synthetic microbiology and metabolic engineering can facilitate the design of microbes/enzymes to catalytically upcycle plastics into the desired products.

## CONFLICT OF INTEREST STATEMENT

The authors declare no conflicts of interest.
